# Potential protective effects of continuous anterior chest compression in the acute respiratory distress syndrome: physiology of an illustrative case

**DOI:** 10.1186/s13054-021-03619-0

**Published:** 2021-06-01

**Authors:** Guillaume Carteaux, Samuel Tuffet, Armand Mekontso Dessap

**Affiliations:** 1 Assistance Publique- Hôpitaux de Paris, Service de Réanimation Médicale, CHU Henri Mondor-Albert Chenevier, 51, Avenue du Maréchal de Lattre de Tassigny, 94010 Créteil Cedex, France; 2 Groupe de Recherche Clinique CARMAS, Université Paris Est-Créteil, 94010 Créteil, France; 3 Institut Mondor de Recherche Biomédicale INSERM 955, 94010 Créteil, France

**To the Editor**,

Continuous anterior chest compression (CACC) may have protective effects in patients with the Acute Respiratory Distress Syndrome (ARDS) by decreasing the anterior chest wall compliance, thus decreasing the anterior transpulmonary pressure and the resulting risk of overdistension [1] along with promoting redistribution of ventilation through the dependent regions. In some ARDS patients, we have even observed an unexpected dramatic improvement in respiratory system compliance while compressing the anterior chest wall. We herein report the physiology of an illustrative case.

## Patient

A 63-year-old male renal transplant recipient with no prior respiratory history was intubated for a SARS-CoV-2 related moderate ARDS. His respiratory mechanics progressively worsened, with a respiratory system compliance below 15 mL/cmH_2_O, a driving pressure of 28 cmH_2_O and appearance of a positive stress index [2] despite a decrease in tidal volume at 5 ml/kg of predicted body weight and the use of a low level of PEEP (6 cmH_2_O). Compression of the anterior chest wall resulted in a disappearance of the stress index pattern and a significant decrease in plateau pressure, prompting further assessment.

## Assessment

The patient was already sedated and paralyzed, in zero degree supine, under assist-control ventilation with the following settings: tidal volume: 350 ml, respiratory rate: 35 breaths/min. We assessed the respiratory mechanics (using flow, airway and esophageal pressure, and elastic pressure–volume curves) and ventilation distribution (using electrical impedance tomography, EIT) at the basal level of PEEP (PEEP6) without or with concomitant CACC (PEEP6 + CACC), and at zero end expiratory pressure (ZEEP). The CACC was achieved by compressing a saline bag on the sternum. The pressure inside the bag was measured and maintained at 80 cmH_2_O by strapping a rigid plate over it following a patented method (WO2019/048774A1).

## Results

**Fig. 1:**
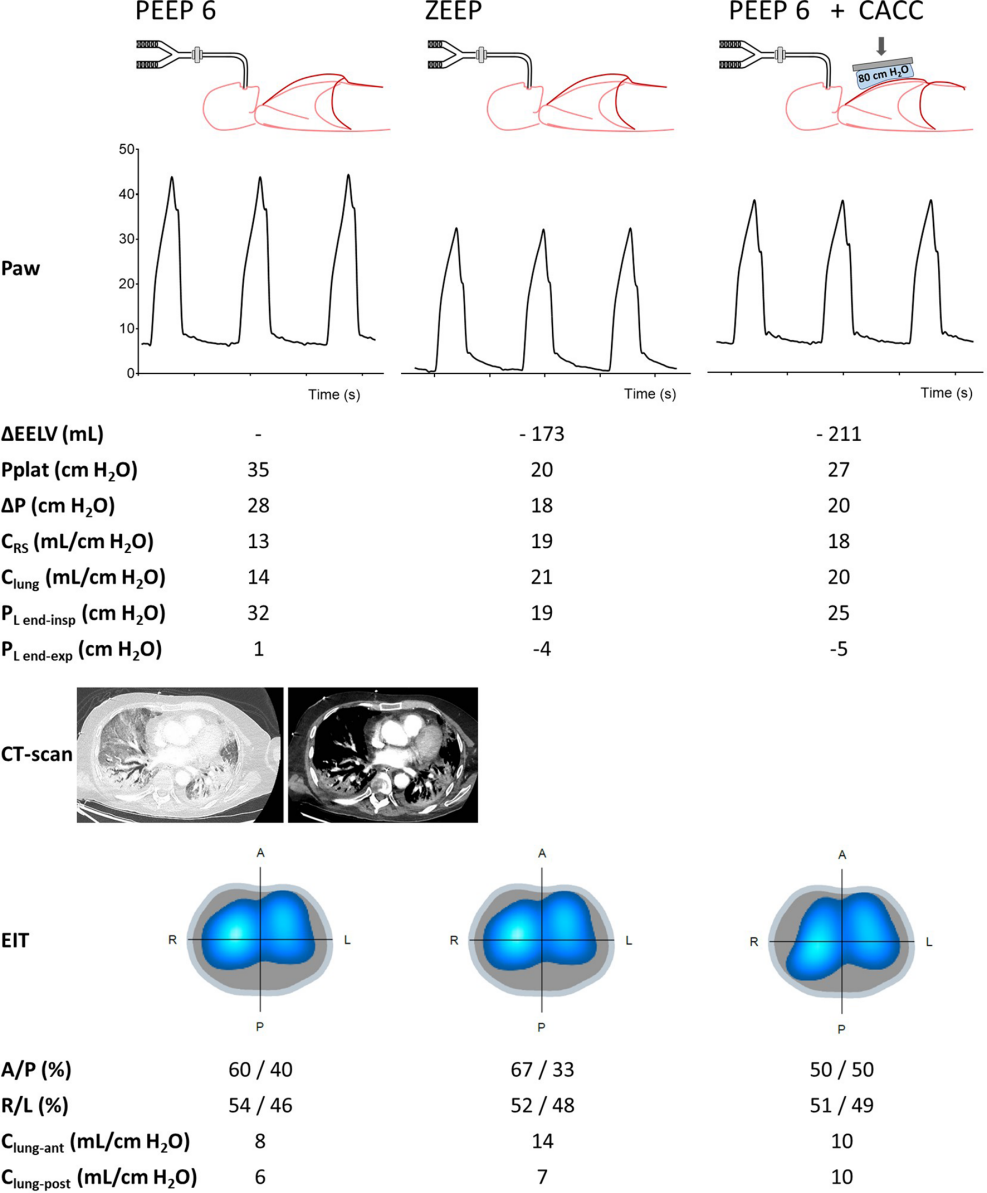
Effect of continuous anterior chest compression on respiratory mechanics and lung aeration. The patient was ventilated in assist control ventilation with a tidal volume of 350 ml in three different conditions: PEEP 6: with a PEEP level of 6 cm H_2_O; ZEEP: with zero end expiratory pressure; PEEP 6 + CACC: with a PEEP level of 6 cm H_2_O and a concomitant continuous anterior chest compression by the mean of a positive pressure of 80 cm H_2_O applied on the anterior chest wall. Paw: airway pressure waveform; ΔEELV: variation of end expiratory lung volume as compared to the “PEEP 6” condition; Pplat: plateau pressure; ΔP: driving pressure defined as the difference between plateau pressure and total PEEP; C_RS_: respiratory system compliance; C_lung_: lung compliance; P_Lend-insp_: transpulmonary pressure at end inspiration, computed as follows: P_Lend-insp_ = Pplat x (E_L_/Ers) where E_L_ is the lung elastance and Ers the respiratory system elastance [5]; P_Lend-exp_: transpulmonary pressure at end expiration, computed as follows: P_Lend-exp_ = PEEPt – P_ESend-exp_ where PEEPt is the total PEEP and P_ESend-exp_ is the end expiratory esophageal pressure value; the chest CT-scan retrieved posterior consolidations with diffuse ground glass opacities, reticulations, and traction bronchiectasis suggestive of a fibrotic evolution; EIT: electrical impedance tomography (Enlight 1800, Timpel, Sao Paulo, Brazil); A/P: percentage of variation in impedance during ventilation (ΔZ) in the anterior (A) and posterior (P) half of the lung; R/L: percentage of variation in impedance during ventilation (ΔZ) in the right (R) and left (L) half of the lung; C_lung-ant_: regional lung compliance in the anterior (ventral) half of the lung; C_lung-post_: regional lung compliance in the posterior (dorsal) half of the lung

Switching from PEEP6 to ZEEP induced a disappearance of the stress index and an increase in respiratory system compliance, mainly related to an increase in regional anterior compliance (Fig. 1). However, in the meantime, EIT evidenced a derecruitment of posterior areas, and the SpO_2_ dropped from 97 to 85%.

**Fig. 2:**
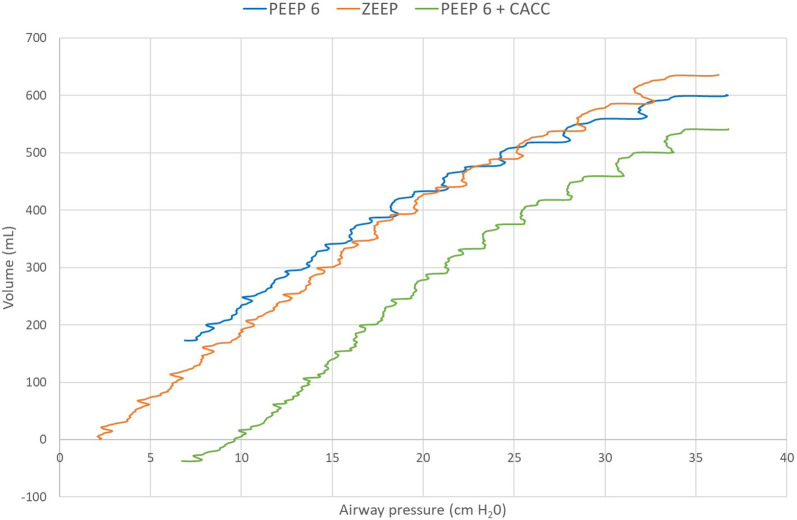
Elastic pressure–volume curve. Elastic pressure–volume curve was obtained by performing a low-flow (4 L/min) inflation up to 35 cm H_2_O of airway pressure in three different conditions: PEEP 6: with a PEEP level of 6 cm H_2_O; ZEEP: with zero end expiratory pressure; PEEP 6 + CACC: with a PEEP level of 6 cm H_2_O and a concomitant continuous anterior chest compression by the mean of a positive pressure of 80 cm H_2_O applied on the anterior chest wall

As compared to PEEP6, the overall effects of PEEP6 + CACC were an increase in pulmonary and respiratory system compliance resulting in a marked decrease in plateau pressure, a slight increase in SpO_2_ to 98%, and a decrease in the end expiratory lung volume (Fig. 1 and 2). Regional effects of CACC were the followings:

- A decrease in anterior (ventral) lung regions distension: the positive stress index pattern disappeared, the end-inspiratory transpulmonary pressure decreased and the regional lung compliance in the anterior half increased.
- A recruitment of the posterior (dorsal) lung regions: the number of pixels showing positive ∆Z in the posterior half of EIT matrix increased by 10% and the regional lung compliance in the posterior half increased.
- A homogenization of tidal ventilation: the ratio between ventilation distributions of the anterior and posterior halves went from 60%/40% to 50%/50%.

No noticeable hemodynamic variation was observed.

## Discussion

The dramatic increase in respiratory system compliance during CACC in this ARDS patient may result from several combined mechanisms: 1-In the part of the lung already aerated but subject to intra-tidal overdistension, the noticeable decrease in the end expiratory lung volume resulted in a leftward shift of the pressure–volume curve below the upper inflexion point [3], 2-the concomitant recruitment in the posterior regions resulted in an increase in the number of aerated lung units [4].

Note that CACC may have affected the esophageal pressure and that EIT is useful for characterizing regional volume variations but may lack precision.

This original description prompts further exploration of the tolerance and physiological effects of CACC in ARDS patients.

## Data Availability

The data used and/or analysed during the current assessment are available from the corresponding author on reasonable request.

## Abbreviations

ARDS: Acute respiratory distress syndrome
CACC: Continuous anterior chest compression
EIT: Electrical impedance tomography
PEEP: Positive end expiratory pressure
ZEEP: Zero end expiratory pressure
